# Multiple Motor Learning Strategies in Visuomotor Rotation

**DOI:** 10.1371/journal.pone.0009399

**Published:** 2010-02-24

**Authors:** Naoki Saijo, Hiroaki Gomi

**Affiliations:** 1 NTT Communication Science Laboratories, Nippon Telegraph and Telephone Corporation, Atsugi, Kanagawa, Japan; 2 ERATO Shimojo Implicit Brain Function Project, Japan Science and Technology Agency, Atsugi, Kanagawa, Japan; The University of Western Ontario, Canada

## Abstract

**Background:**

When exposed to a continuous directional discrepancy between movements of a visible hand cursor and the actual hand (visuomotor rotation), subjects adapt their reaching movements so that the cursor is brought to the target. Abrupt removal of the discrepancy after training induces reaching error in the direction opposite to the original discrepancy, which is called an aftereffect. Previous studies have shown that training with gradually increasing visuomotor rotation results in a larger aftereffect than with a suddenly increasing one. Although the aftereffect difference implies a difference in the learning process, it is still unclear whether the learned visuomotor transformations are qualitatively different between the training conditions.

**Methodology/Principal Findings:**

We examined the qualitative changes in the visuomotor transformation after the learning of the sudden and gradual visuomotor rotations. The learning of the sudden rotation led to a significant increase of the reaction time for arm movement initiation and then the reaching error decreased, indicating that the learning is associated with an increase of computational load in motor preparation (planning). In contrast, the learning of the gradual rotation did not change the reaction time but resulted in an increase of the gain of feedback control, suggesting that the online adjustment of the reaching contributes to the learning of the gradual rotation. When the online cursor feedback was eliminated during the learning of the gradual rotation, the reaction time increased, indicating that additional computations are involved in the learning of the gradual rotation.

**Conclusions/Significance:**

The results suggest that the change in the motor planning and online feedback adjustment of the movement are involved in the learning of the visuomotor rotation. The contributions of those computations to the learning are flexibly modulated according to the visual environment. Such multiple learning strategies would be required for reaching adaptation within a short training period.

## Introduction

People have an ability to adapt their body movement to external environments. When the visually perceived hand position is displaced from the actual hand position by a prism or computer device, visually guided reaching is initially disturbed but recovers after training [1]–[6]. Aftereffects, movement errors generated by unexpectedly removing the displacement of the visual hand position after training, suggest that the central nervous system (CNS) learns a new transformation from the visual input to motor output.

Recent studies [7], [8] have shown that training with a gradually increasing visuomotor discrepancy results in a larger aftereffect than training with a suddenly introduced one. Although the aftereffect difference would indicate a difference in the new transformation the CNS has learned, it is still unclear whether the learned visuomotor transformations are qualitatively different between the training conditions.

Previous studies of visually guided reaching, on the other hand, have suggested that the reaching movement is controlled by both feedforward and feedback motor commands [9]–[14]. The movement is prepared from the visual information of the target (motor planning) and the feedforward motor command is subsequently generated from the desired movement via an internal model of the arm and the environment (feedforward control) [11], [13], [14]. After the hand movement is initiated, the feedback motor command is generated using the sensory (e.g., visual) feedback signal associated with the ongoing movement in order to adjust the movement in mid-flight (feedback control) [9], [10], [13]. When learning a new visuomotor transformation, the CNS could update the feedforward and/or feedback motor commands to reduce the movement error. For example, the ongoing movement can be corrected using the visual feedback information and/or the pre-planed hand movement can be modified in the next trial. Therefore, the CNS may have several strategies for learning the new visuomotor transformation. The difference in selected learning strategy between the training conditions could result in the aftereffect difference.

Here, we investigate the qualitative difference in the visuomotor learning process between the different training conditions. Subjects were exposed to a sudden or gradual visuomotor rotation and practiced the reaching movement. To examine the contribution of the online visual feedback control to the learning of the visuomotor rotation, we investigated not only the changes in hand movement due to the learning of the visuomotor rotation but also the changes in responses to transient visual perturbations. The results indicate that the learning strategy is flexibly changed according to the training condition to reduce the reaching error in the short-training period. This strategy change leads to the difference in the aftereffect. Parts of the experimental data shown in this study have been preliminary reported elsewhere [15].

## Materials and Methods

### Subjects

Eighteen subjects (11 males, 7 females; 21 to 38 years old, average 28.2±5.0 years old) participated in experiment 1, and fifteen subjects (7 males, 8 females; 20 to 31 years old, average 25.4±4.2 years old) participated in experiment 2. All subjects had normal or corrected to normal vision, and all were right-handed. None of the subjects had ever experienced any visual or motor deficits. All subjects gave written informed consent to participate in the study, which was approved by the NTT Communication Science Laboratories Research Ethics Committee.

### Apparatus

Each subject sat in front of the manipulandum [16] while strapped securely to the chair back with the head placed on a chin support. The right forearm was tightly coupled to the handle with a molded plastic cuff and supported against gravity by a horizontal beam. The manipulandum system was digitally controlled to reduce the dynamical effect of the handle on the subject's hand. Therefore, subjects were able to move the handle easily in any direction.

Visual stimuli were generated by a computer and projected by a data projector (refresh rate, 60 Hz; PLUS U2-X2000; PLUS Vision Corp.,Tokyo, Japan) on a horizontal screen (1.2×1.0 m) placed just above the subject's forearm. The screen concealed the arm from the subject's view. The start position (blue circle, 1 cm in diameter), target (green disk, 2 cm in diameter) and cursor (red disk, 1 cm in diameter) were shown on the screen. The cursor position was aligned just above the hand position, while its position was rotated around the reaching start position in the experiments. The computer received the hand position measured by the manipulandum system at 2 kHz in real time and updated the cursor position at 60 Hz (which corresponded to the refresh rate of the data projector). The update timing of the cursor position on the screen was directly measured with a photodiode (Hamamatsu Photonics S1223-1) at 3 kHz. The time delay from reception of the hand position to the cursor position update was 33.3±6.1 ms (mean ± SD). The hand position was recorded for a duration of 3.0 s at 500 Hz by the manipulandum system. The recording started 0.3 s before target was shown.

### Experimental Protocol

**Figure 1:**
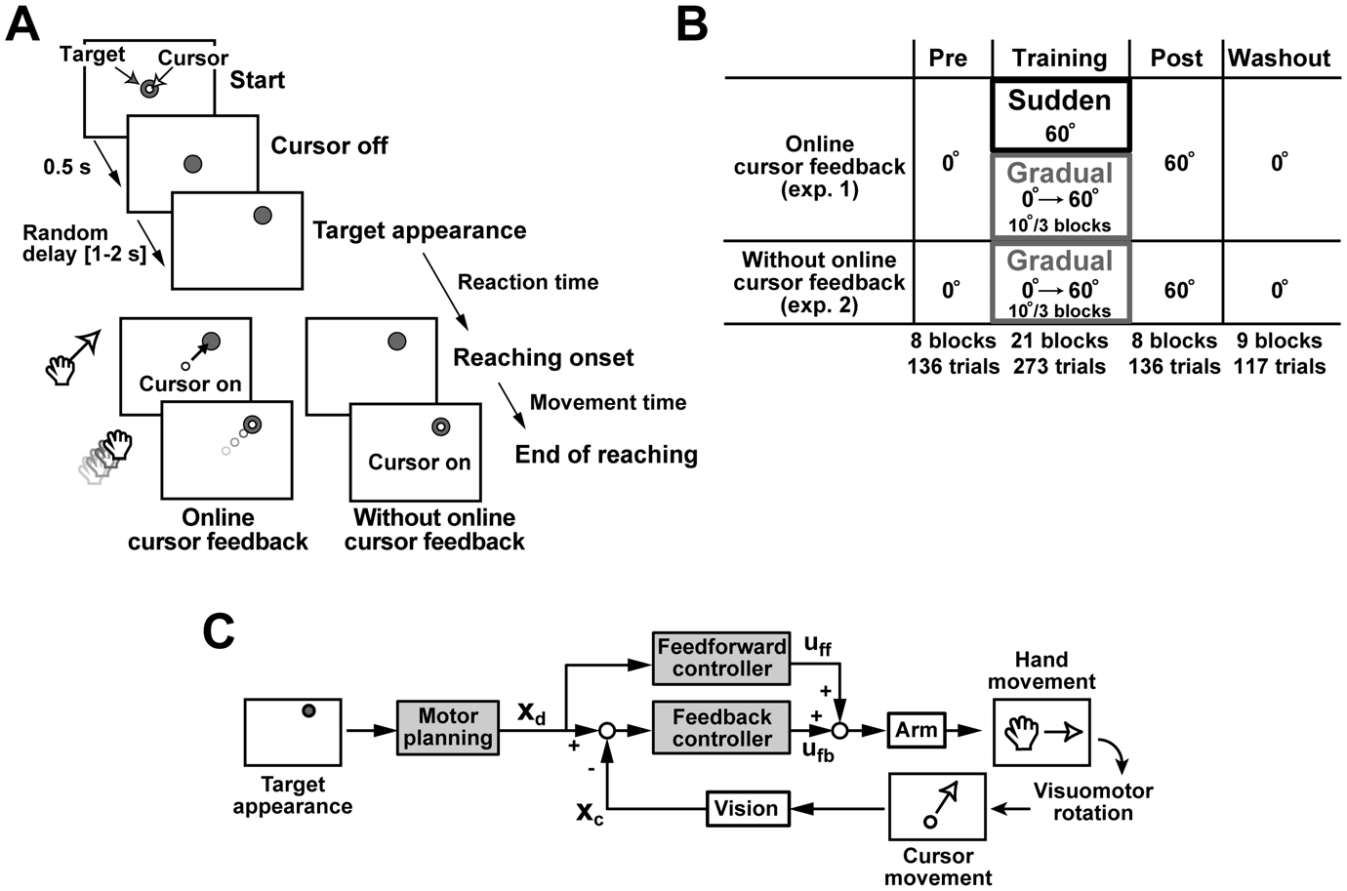
Experimental procedure and computational scheme for visually guided reaching. **A**, Time sequence of one trial. There were two conditions: reaching with and without online cursor feedback. See Materials and Methods for details. **B**, Total trial procedure. The angles are the visuomotor rotation angles. Each experiment consisted of four phases: pre-training, training, post-training, and washout phases. The cursor online feedback was shown in experiment 1 but not in experiment 2. In experiment 1, subjects were divided into two groups and the visuomotor rotation was suddenly or gradually introduced in the training phase for each group. The 60° CCW visuomotor rotation was continued in the post-training phase and then suddenly changed to 0° in the washout phase. **C**, Computational scheme for visually guided reaching, where 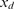 represents desired movement of the arm, 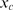 indicates feedback information of current movement, and 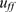 and 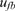 indicate feedforward and feedback motor commands, respectively. This scheme assumes that target presentation triggers the motor planning and that the feedforward controller transforms the desired movement into motor commands. The manipulandum system converts the hand movement into cursor movement in the rotationally biased direction on the screen. The feedback controller adjusts the ongoing movement using the feedback information of the cursor movement.

Figure 1A shows the temporal sequence of stimuli and behavioral events. The hand cursor, start position, and the target were initially shown on the screen. The start position and the target were placed at (−0.15, 0.45), where (*x*, *y*) indicates *x* (in meters) in the rightward and *y* (in meters) in the forward direction relative to the shoulder position on the work plane. After the cursor position had been aligned with the start position for 0.5 s, the start position and the cursor turned off with a beeping sound. Following a random delay period (1.0–2.0 s), the target was shown at one of twelve positions around the start position with intervals of 30° on the screen. The target positions were 15 cm from the start position. Here, the rightward direction of the target position was 0° and the counterclockwise (CCW) direction was positive. This appearance of the target was the cue for reaching initiation. Subjects were instructed to move the cursor to the target as soon and as accurately as possible immediately after the target was shown. They were also asked to fixate on the start position during the movement to avoid the effect of eye movement on the reaching movement and told not to try to predict the target appearance time and its position, since both were randomized.

In the cursor feedback trial, the cursor was shown on the screen during the trial in the following manner. In the first experiment (experiment 1), the cursor was turned on immediately after the reaching onset and illuminated for 1.0 s (“online cursor feedback” in Fig. 1A). A short beep was given 2.7 s after the target appearance (which corresponds to the end of the recording time) and the cursor was again turned on for 1.0 s to indicate the reaching end position and the end of the trial. In the second experiment (experiment 2), the cursor feedback was eliminated during the reaching movement, but the cursor was turned on at the end of the reaching and illuminated until the end of the trial (“without online cursor feedback” in Fig. 1A). The methods for detecting the reaching onset and end are explained in the Data analysis section. In both experiments, the cursor position relative to the hand position was rotated CCW around the start position. This transformation is referred to as visuomotor rotation. The subjects were not informed of the presence of the visuomotor rotation. After the end of one trial, the target and cursor were turned off and the hand was automatically pulled back to around the start position by the manipulandum.

Additionally, two other types of probe trials were presented: cursor perturbation trials and catch trials. In the cursor perturbation trial, the cursor movement direction suddenly changed during the reaching movement. The cursor was turned on with the visuomotor rotation immediately after the reaching onset (visible for 1.0 s), and then its movement direction began to be modified (+20° or −20° rotation around the cursor position at the perturbation onset) 150 ms after the cursor onset. The purpose of this perturbation was to induce a mid-flight adjustment of the hand movement [17]. A short beep indicating the end of the trial was given 2.7 s after the target had appeared, but unlike in the cursor feedback trial, the cursor was not turned on again in order to avoid adaptation to the additional coordinate transformation caused by the cursor perturbation. During catch trials, the cursor was *not* turned on. The beeping sound was provided 2.7 s after the target appearance to signal the end of the trial. After the end of the cursor perturbation and catch trials, the target was turned off and the hand was automatically pulled back to around the start position by the manipulandum.

Each experiment consisted of four trial-phases: a pre-training phase, training phase, post-training phase, and washout phase (Fig. 1B). Subjects were given a break of 15 to 20 minutes between the phases to avoid fatigue.

The pre-training phase consisted of eight blocks of 17 trials (total 136 trials). One block consisted of 12 cursor feedback trials (one trial for each target direction), four cursor perturbation trials (two trials for each perturbation direction), and one catch trial. The order of the cursor feedback and cursor perturbation trials was randomized within each block. The catch trials were presented at the end of each block. The cursor perturbation and catch trials were introduced with a 0° target. The angle of the visuomotor rotation was 0°.

The training phase consisted of 21 blocks of 13 trials (total 273 trials) in which the visuomotor rotation was provided. Each block consisted of 12 cursor feedback trials and one catch trial. The cursor feedback trials were presented in random order in each block. The catch trials were presented with a 60° target at the end of each block. The visuomotor rotation angle was 0° in the first three blocks. In the fourth block, it was suddenly changed to 60° and sustained for the remaining 18 blocks (sudden condition) or was changed by 10° every three blocks and the rotation angle in the last three blocks was kept at 60° (gradual condition).

The post-training phase consisted of eight blocks of 17 trials (total 136 trials). Each block consisted of 12 cursor feedback trials, four cursor perturbation trials, and one catch trial, as in the pre-training phase. The 60° visuomotor rotation was provided throughout this phase. The cursor perturbation and catch trials were introduced with the 60° target to align the ideal hand movement directions in those trials with those in the pre-training phase.

The washout phase consisted of nine blocks of 13 trials (117 trials). Each block consisted of 12 cursor feedback trials and one catch trial, as did the training phase. The catch trials were introduced with a 0° target. The 60° visuomotor rotation was introduced in the first three blocks and then the rotation angle was suddenly changed to 0° from the fourth block and sustained for the remaining six blocks.

Eighteen subjects participated in experiment 1 and were divided into two groups of nine in the training phase. One group was trained on the 60° visuomotor rotation in the sudden condition and the other group was trained in the gradual condition. The other 15 subjects participated in experiment 2 and were trained in the gradual condition without the online cursor feedback.

### Data Analysis

The recorded hand position was filtered (fourth-order Butterworth filter; 15–Hz cutoff frequency) to remove the high-frequency components. The hand velocity and acceleration were computed by numerically differentiating the position data.

The reaching onset was detected when the tangential acceleration exceeded 0.5 m/s^2^. The end of the reaching was defined by a method similar to that in Imamizu et al [3]. We calculated the two-dimensional curvature and detected when it exceeded 0.1 m^−1^ after the tangential velocity had exceeded 0.2 m/s after the movement onset. We defined this timing as the end of the reaching. Note that, in the condition without the online cursor feedback (experiment 2), the timing of the reaching end was calculated by this method in real time and the cursor activation coincided with this timing.

We quantified the following eight characteristics indices of reaching movement: reaction time (RT), movement time (MT), peak velocity (PV), time to peak velocity (Tpv), initial direction error (I-DE), endpoint direction error (E-DE), trajectory curvature (C), and endpoint distance (ED).

The RT was defined as the time difference between the target presentation and the reaching onset. The timings of target presentation were measured with the photodiode. The MT was defined as the temporal duration between the reaching onset and the end of the reaching. The PV was defined as the peak tangential velocity during the movement. The Tpv was calculated as the time difference between the movement onset and the time at the PV. The I-DE was obtained by the directional difference between the target direction from the start position and the direction of the cursor velocity vector 100 ms after the movement onset. The positive error indicates error in the CCW direction. The E-DE was obtained from the directional difference between the target direction and the direction of the reaching end position from the start position. Here, we considered that the cursor was successfully moved in the correct direction if the E-DE was within the range of ±5.7° (success margin). This margin was set to the summation of the sizes of the reaching target and the cursor placed in the vicinity of the target. The C was quantified by subtracting the E-DE from the I-DE. The ED was defined as the distance between the start position and the reaching end position. Note that, although the cursor was not shown in the catch trials, the I-DE, E-DE, C and ED were obtained as if it had been.

In the cursor perturbation trial, the hand response to the perturbation was analyzed. The hand-movement data were aligned at the time of the perturbation onset, which corresponded to 150 ms after the cursor appearance. Hand response latency was detected when a significant difference between the hand accelerations for the positive (+20°) and negative (−20°) perturbations was detected by *t* test (5%) at each data sampling time after the perturbation onset. The response amplitude was calculated by taking the temporal average of *y*-acceleration difference between the positive and negative perturbations for the interval of 250–300 ms from the perturbation onset. This time interval was defined by the response latency (see Results).

For the statistical analysis, Student's paired and unpaired *t* tests were used for comparison between the two groups. To determine the effects of the training condition (among-subjects factor) and the training phase (within-subjects factor) on each index, we performed a two-way mixed design ANOVA followed by a *post hoc* paired or unpaired *t* test with Bonferroni's correction. The significance level was set at *p*<0.05.

### Computational Scheme for Visually Guided Reaching

To explain the behavioral difference among the training conditions, we here postulate a computational scheme for visually guided reaching and then investigate the relationship between the computations and the above-mentioned experimental variables.

As mentioned in the Introduction, visually guided reaching is controlled by both feedforward and feedback motor commands. Here, the feedforward motor command refers to the motor command unchanged by the sensory (e.g. visual) feedback signal associated with the ongoing movement, whereas the feedback motor command refers to that based on the online sensory feedback information.

As previous studies [11], [18] have suggested, we considered that the generation process of the feedforward motor command consists of two stages. The first stage is the computation of desired movement preparation from the visual target information, which would be finished before the initiation of the actual hand movement. Here we refer to this computation as motor planning. The next stage is the process for generating the final motor command from the desired movement via the internal model of the arm and the environment, which is executed for not only the movement initiation but also during the entire movement. We refer to this computation as feedforward control. Note that, the feedforward control is widely employed in robotics [19] and computational neuroscience [9], [11], [14], [20]–[23] fields, and those computations have been experimentally examined for biological motor controls [24]–[30]. To generate the feedback motor command, the feedback controller uses the sensory feedback signal associated with the ongoing movement [10], [17]

The computational scheme for the reaching movement is outlined in Fig. 1C. The desired movement 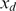 is generated by the motor planning and transformed into the feedforward motor command 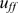 by the feedforward controller. The movement error is calculated from the current hand position 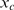
informed by the online visual feedback and is transformed into the feedback motor command 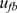 via the feedback controller.

To examine which computation is changed by the leaning, we need to make a link between the experimental variables and the computations. The RT may reflect the processing time for the motor planning and the generation of the initial feedforward motor command. The I-DE is inherently related to the initial feedforward motor command, since it was obtained before the ongoing-movement corrections based on the visual feedback were induced. Here, we need to categorize the I-DE changes observed during the leaning into two types. One is concomitant with RT change and the other is not concomitant with RT change. Since the processing time for the motor planning might be involved in the RT, it would be reasonable to postulate that the former is related to the computation change for planning and the latter is related to the feedforward-controller modification. The E-DE, ED, MT, PV, and Tpv, defined in the Data analysis section, are related to both feedforward and feedback control. The response to the cursor perturbation is assumed to be generated by the feedback control. The movement in the catch trials is considered to be mostly generated by the feedforward controller, since the visual feedback, which was available in the most of the trials, was unexpectedly eliminated.

## Results

### Sudden or Gradual Visuomotor Rotation Learning with Online Cursor Feedback

#### Trajectory changes in two rotation conditions

**Figure 2:**
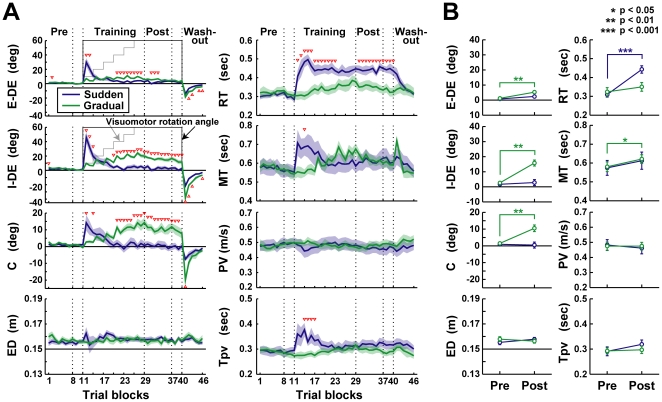
Changes in reaching movement characteristics by the sudden and gradual visuomotor rotation learning. **A**, Mean characteristics indices of reaching movement across subjects as a function of trial block in experiment 1. Thick blue and green lines in each panel indicate the sudden and gradual conditions, respectively. The visuomotor rotation angles (black and gray thin line for the sudden and gradual conditions, respectively) are superimposed on the panels of the E-DE and I-DE. Shaded areas represent the SE. Open red triangles indicate the trial blocks in which the indices were significantly different between the sudden and gradual conditions (*t* test, *p*<0.05). **B**, Changes in the averaged indices between pre- and post-training phases. In all panels, the asterisks (*, **, and ***) denote the significance of differences: *p*<0.05, *p*<0.01, and *p*<0.001, respectively. Error bars represent the SE.

To assess the changes in the reaching movements associated with the learning of the visuomotor rotation, we quantified the eight characteristics indices (RT, MT, PV, Tpv, I-DE, E-DE, C, and ED) of the reaching movement. Figure 2A displays the mean indices across subjects as a function of trial block. Each index was averaged in each trial block. In the sudden condition (blue lines), the trajectory errors (the E-DE, I-DE, and C) largely increased, with small changes in the ED, immediately after the 60° rotation had been applied. Although the ED slightly fluctuated, there was no significant difference between the conditions (*t* test, *p*>0.07). The RT, MT, and Tpv increased, with slightly decreasing PV, at the beginning of the training phase. Then the trajectory errors, MT, PV and Tpv approximately returned to the baseline as the training was continued. However, the RT did not decrease until the post-training phase.

In the gradual condition (green lines in Fig. 2A), on the other hand, the indices did not change abruptly when the visuomotor rotation was applied. The E-DE was always small until the post-training phase. However, the I-DE and C increased progressively as the training was continued, indicating that the hand trajectory was curved by the training. At the same time, the MT also increased continuously, suggesting that the curved trajectory might lead to the increase in MT.

In both conditions, negative aftereffects were observed. When the 60° rotation was suddenly removed in the washout phase, the E-DE, I-DE, and C changed to negative values. Note that the MT in the gradual condition increased abruptly with the increase in the C in the first block of the washout phase, suggesting that the curved trajectory causes the prolonged MT. The I-DE and E-DE in the gradual condition were significantly larger than those in the sudden condition in four of six blocks in the washout phase (*t* test, *p*<0.05; the red triangles in Fig. 2A), indicating that the aftereffect was larger in the gradual condition, which is consistent with the previous study [7].

To compare the changes in those indices between the pre- and post-training phases and between the training conditions, we obtained the averaged indices in those phases (Fig. 2B). A two-way mixed design ANOVA revealed a significant interaction effect between the training phase (pre- or post-training phase) and the training condition (sudden or gradual condition) on the E-DE (*F*_(1,16)_  = 4.60, *p*<0.05), I-DE (*F*_(1,16)_  = 18.70, *p*<0.001), C (*F*_(1,16)_  = 13.11, *p*<0.01), ED (*F*_(1,16)_  = 5.44, *p*<0.05), and RT (*F*_(1,16)_  = 15.55 *p*<0.001). A *post hoc* test showed that in the sudden condition, the RT in the post-training phase was significantly longer than that in the pre-training phase (paired *t* test, *p*<0.001, top right panel in Fig. 2B). In contrast, in the gradual condition, it was not significantly different between the pre- and post-training phases (paired *t* test, *p* = 0.14) but the I-DE and C were significantly larger in the post- than the pre-training phases (paired *t* test, *p*<0.01, second and third row of left panels in Fig. 2B). Although the mean E-DE in the post-training phase of the gradual condition was significantly larger than that in pre-training phase (paired *t* test, *p*<0.05), the mean E-DE in the post-training phase was 5.25±0.63° (mean ± SE across subjects) and within the reaching success margin (±5.7°, see Data analysis). Therefore, we considered that the cursor reached the target position after the training.

The ANOVA showed no significant interaction effects on the MT (*F*_(1,16)_  = 0.01), PV (*F*_(1,16)_  = 0.73), and Tpv (*F*_(1,16)_ = 2.98), but showed the significant main effect of the training phase on the MT (*F*_(1,16)_  = 8.10, *p*<0.05) and Tpv (*F*_(1,16)_  = 5.60, *p*<0.05). A *post hoc* test revealed that in the gradual condition, the MT in the post-training phase was significantly longer than that in the pre-training phase (paired *t* test with Bonferroni's correction, *p* = 0.0109), but not in the sudden condition (*p* = 0.16). A significant difference in the Tpv was not found by the *post hoc* test (*p*>0.05). The MT increase with no change in the Tpv in the gradual condition indicates that the later phase of the movement (e.g., deceleration phase) is prolonged. Additionally, the increase of MT after the training was correlated with that of C (*r* = 0.84, *p*<0.01). Therefore, the MT elongation would be caused by the curved trajectory in the late phase of the movement.

#### Catch-trial effects

There are two possible reasons the trajectory was curved after the learning in the gradual condition. It was either adjusted in mid-flight by the online cursor feedback information, or generated just in a feedforward manner. If the latter is the case, the subjects should have been able to reach the target correctly even in the catch trials, in which the cursor was not shown during the trial.

**Figure 3:**
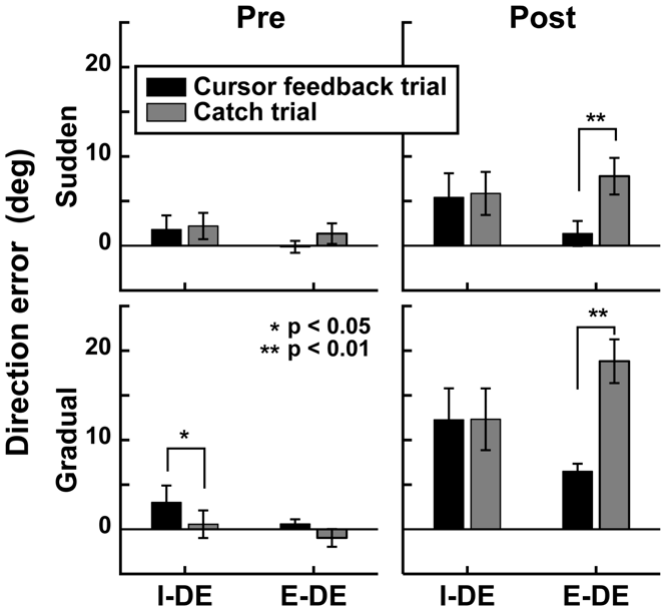
Catch trial effects. Each panel shows the mean I-DEs and E-DEs of cursor feedback trials (black bars) and catch trials (gray bars) across subjects. The asterisks (* and **) denote the significance of differences: *p*<0.05 and *p*<0.01, respectively. Error bars denote the SE. Left and right panels indicate the pre- and post-training phases, respectively. Top and bottom panels indicate the sudden and gradual conditions, respectively. Note that the cursor-feedback trials with the same visual target as the catch trials were selected to calculate the mean I-DE and E-DE.

Figure 3 displays the I-DE and E-DE of the cursor-feedback and catch trials in the pre- and post-training phases averaged across subjects. Note that, since the mean I-DE and E-DE of the cursor-feedback trials were calculated by selecting the trial with the same visual target as the catch trial (0° and 60° in the pre- and post-training phases, respectively), we could assume that the observed differences were caused by the difference in the trial type, not by the difference in the reaching direction.

In the pre-training phase (left panels in Fig. 3), the I-DE and E-DE were not significantly different between the cursor-feedback and catch trials (paired *t* test, *p*>0.05), with the exception of the I-DE in the pre-training phase of the gradual condition (*p*<0.05). Note that, since this I-DE difference was small (2.1±0.7°) and observed before the learning of the visuomotor rotation, it was assumed to be caused by the trial variance.

In the post-training phase (right panels in Fig. 3), on the other hand, the E-DEs in the catch trials were significantly larger than those in the cursor-feedback trials for both conditions (paired *t* test, *p*<0.01), while the I-DEs were not (*p*>0.5), indicating that the online cursor feedback was used for accurate reaching after the training. Additionally, the E-DE of the catch trials in the gradual condition was significantly greater than that in the sudden condition (*t* test, *p*<0.01), suggesting that the online cursor feedback contributed to reducing the directional error in the gradual condition more than in the sudden condition. This indicates that the curved trajectory in the gradual condition was not generated in a feedforward manner, whereas the online cursor feedback was required in order to adjust the reaching direction after the training in the gradual condition.

#### Cursor perturbation effects

**Figure 4:**
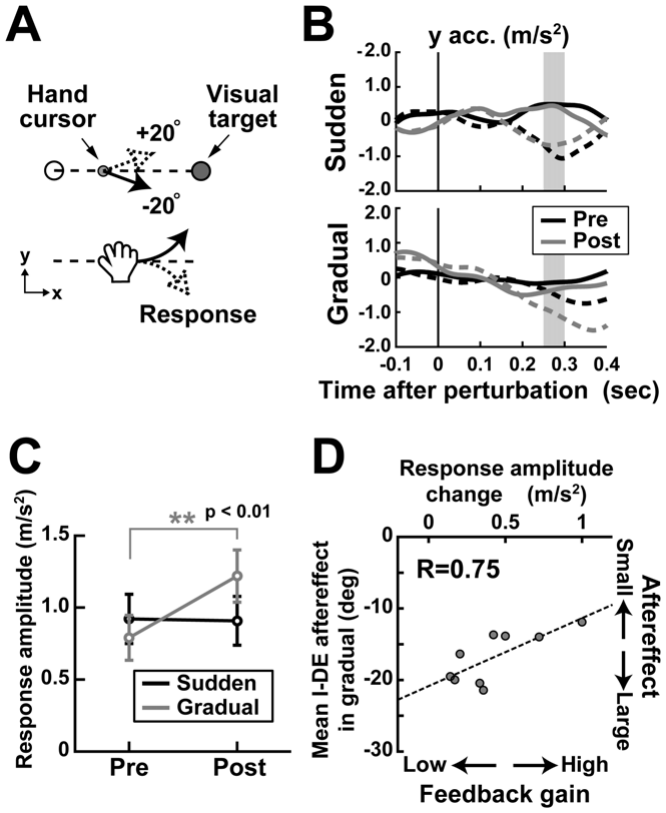
Cursor perturbation effects. **A**, Cursor perturbation. The cursor movement direction relative to the hand movement direction was suddenly shifted ±20° from 150 ms after the cursor was turned on. **B**, Averaged acceleration patterns in the *y*-direction for the negative (solid) and positive (dashed) direction perturbations. Time zero corresponds to the onset of the cursor perturbation. **C**, Mean amplitude of acceleration response across subjects. Error bars denote the SE. The response amplitude was calculated from the temporal average of the *y*-acceleration difference between the perturbation directions for the interval of 250–300 ms from the perturbation onset (shaded areas in B). The double asterisks denote the significance of differences (*p*<0.01). **D**, The relationship between the I-DE aftereffect and the response amplitude change in individual subjects in the gradual condition. The I-DE was averaged from 41^st^ to 46^th^ blocks for each subject. The negative I-DE indicates a large aftereffect. The positive value of response amplitude change indicates the increase in the response amplitude to the cursor perturbation after the training, which corresponds to the increase in the feedback gain due to the learning of the gradual visuomotor rotation.

To compare the contribution of the online visual feedback control to the learning of the visuomotor rotation between the training conditions, we estimated the gain of the visual feedback control by analyzing the hand response to the cursor perturbation (Fig. 4A). The hand acceleration along the *y*-axis changed soon after the cursor perturbation was applied (Fig. 4B). The negative direction perturbation accelerated the hand in the positive *y*-direction (solid curve) and vice versa (dashed curve). For the gradual-condition subject, the difference in hand *y*-directional acceleration between the positive and negative perturbations was clearly larger in the post-training phase than in the pre-training phase (gray lines), while it appears to be slightly smaller for the sudden-condition subject.

To compare the response amplitudes between the pre- and post-training phases, we calculated the differences between the *y*-acceleration patterns for the cursor perturbation in the positive and negative directions and then temporally averaged that difference for the interval of 250–300 ms after the perturbation onset (gray shades in Fig. 4B). This time window was defined by the response latencies to capture the initial phase of the response. The mean response latencies across subjects in the pre- and post-training phases were 202±8 and 216±25 ms in the sudden condition and 228±10 and 213±7 ms in the gradual condition, respectively. The ANOVA revealed no significant difference in these latencies (*F*_(1,16)_  = 0.002 for training phase; *F*_(1,16)_  = 0.92 for training condition; *F*_(1,16)_  = 1.03 for phase × condition).

Figure 4C shows the mean response amplitude across subjects. A two-way mixed design ANOVA revealed a significant interaction effect between the training phase and training condition (*F*_(1,16)_  = 5.69, *p*<0.001). A *post hoc* test indicated that in the gradual condition, the response amplitude in the post-training phase significantly larger than that in the pre-training phase (paired *t* test, *p*<0.01), while that in the sudden condition was not (*p*>0.5). This indicates that the gain of the visual feedback control increased with the learning in the gradual condition. Additionally, this gain increase in the gradual condition was correlated with the size of the I-DE aftereffect. Figure 4D shows the I-DE aftereffects for all subjects in the gradual condition as a function of change in response amplitudes to the cursor perturbation. There is a significant correlation between these indices (*r* = 0.75, *p*<0.05). This indicates that in the gradual condition, the increase in the gain of the visual feedback control for the learning of the visuomotor rotation led to the small I-DE aftereffect.

### Gradual Visuomotor Rotation Learning without Online Cursor Feedback

The results of experiment 1 suggest that the in the learning in the gradual condition, the adaptation of the online visual feedback control is involved, instead of the additional computations before the reaching movement initiation. The next question then is: Is the additional computation involved in the learning of the rotation in the gradual condition if the online visual feedback is not available? In experiment 2, we eliminated the online cursor feedback during the movement with gradually increased visuomotor rotation (Fig. 1A). The cursor was turned on at the end of the reaching movement. Therefore, the reaching movement could not be adjusted in mid-flight using the cursor feedback information.

**Figure 5:**
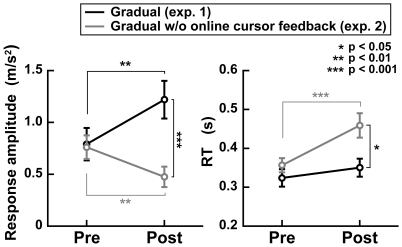
Response amplitudes to cursor perturbation and RTs after learning with and without online cursor feedback. Left and right panels show the mean amplitudes of acceleration responses to the cursor perturbation and the mean RTs for the reaching initiation across subjects, respectively. Black lines indicate the gradual condition with online cursor feedback (exp. 1) and gray lines indicate the gradual condition without online cursor feedback (exp. 2). Note that the data indicated by the black lines are the same as those represented by the gray line in Fig. 4C (response amplitude) and the green line in top-right panel of Fig. 2B (RT). The asterisks (*, **, and ***) denote the significance of differences: *p*<0.05, *p*<0.01, and *p*<0.001, respectively.

We first examined the response to the cursor perturbation as we did in experiment 1. The left panel of Fig. 5 shows the mean response amplitude across subjects in the gradual condition of experiments 1 and 2. A two-way mixed design ANOVA reveals that there was a significant interaction between the training phase (pre- or post-training phase) and the availability of the cursor feedback (experiment 1 or 2) (*F*_(1,21)_  = 28.61, *p*<0.0001). A *post hoc* analysis shows that in experiment 2, the response amplitude in the post-training phase was significantly smaller than that in the pre-training phase (paired *t* test, *p*<0.01), whereas it was larger than the pre-training phases in the gradual condition in experiment 1 (*p*<0.01). Additionally, the response amplitudes in the post-training phase were significantly different between experiments 1 and 2 (unpaired *t* test, *p*<0.001). This indicates that the gain of the visual feedback control decreased after the training the gradual visuomotor rotation without online cursor feedback.

The right panel of Fig. 5 shows the mean RT across subjects in the gradual condition of experiments 1 and 2. A two-way mixed design ANOVA reveals that there was a significant interaction between the training phase and the availability of the cursor feedback (*F*_(1,21)_  = 28.61, *p*<0.0001). A *post hoc* analysis shows that the RT was significantly longer in the post-training phase than in the pre-training phase in experiment 2 (paired *t* test, *p*<0.001), whereas it was not significantly different between the phases in experiment 1 (*p* = 0.14). In addition, the RTs in the post-training phase were significantly different between experiments 1 and 2 (unpaired *t* test, *p*<0.05). These results therefore suggest that if the online visual feedback is not available, the additional computations would be recruited before the reaching movement initiation to learn the gradual visuomotor rotation.

**Figure 6:**
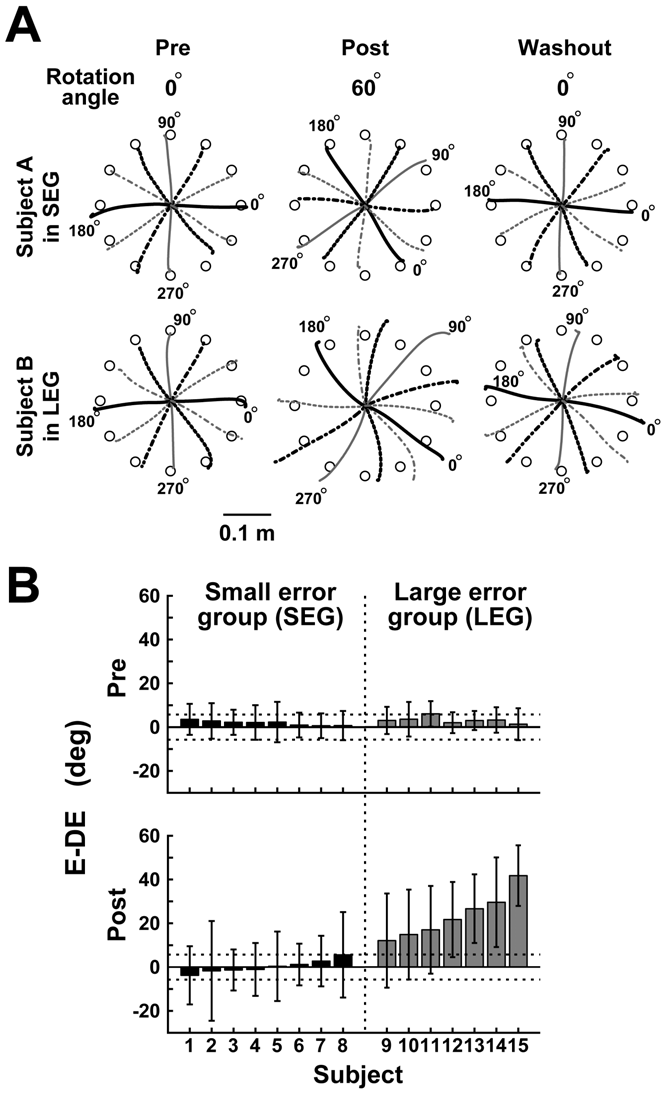
Reaching performances in the gradual visuomotor rotation learning without online cursor feedback. **A**, Averaged hand trajectories of typical subjects in the pre-training (left panels), post-training (middle panels), and washout phases (right panels) of the “without online cursor feedback” condition (experiment 2). Upper and lower panels correspond to the trajectories of typical subjects in the small error group (SEG) and large error group (LEG), respectively. Small white disks in each panel indicate the reaching target locations. The hand was moved from the center outwards. The trajectories are averaged 1 sec from the reaching onset in each reaching direction. The angles placed aside of the endpoint of the trajectories indicate the directions of the visual targets. **B**, Averaged E-DE in pre- and post-training phases for all subjects. Error bars denote the SD. Dashed lines indicate the reaching success margin (±5.7°, see Data analysis).

After training in experiment 2, some subjects were able to accomplish the reaching task and others were not. Figure 6A shows the averaged hand trajectories of two typical subjects in either group. A striking difference in the trajectory between the subjects was found in the post-training phase (second column in Fig. 6A). Subject B (lower panel) was unable to reach the target correctly, while subject A was able to (upper panel). The reaching endpoints of subject B not only overshot the target distance but also rotated less than 60° in the CW direction from the visual target direction, indicating that he did not finish learning the 60° CCW visuomotor rotation completely.

Figure 6B shows the averaged E-DEs in the pre- and post-training phases for all subjects in experiment 2, which are ordered by E-DE in the post-training phase. Here, we divided the subjects into two subgroups according to the averaged E-DEs in the post-training phase: a “small-error group” (SEG, eight subjects) whose E-DEs were within the reaching success margin (see Data analysis) and a “large-error group” (LEG, seven subjects) whose E-DEs exceeded the margin.

**Figure 7:**
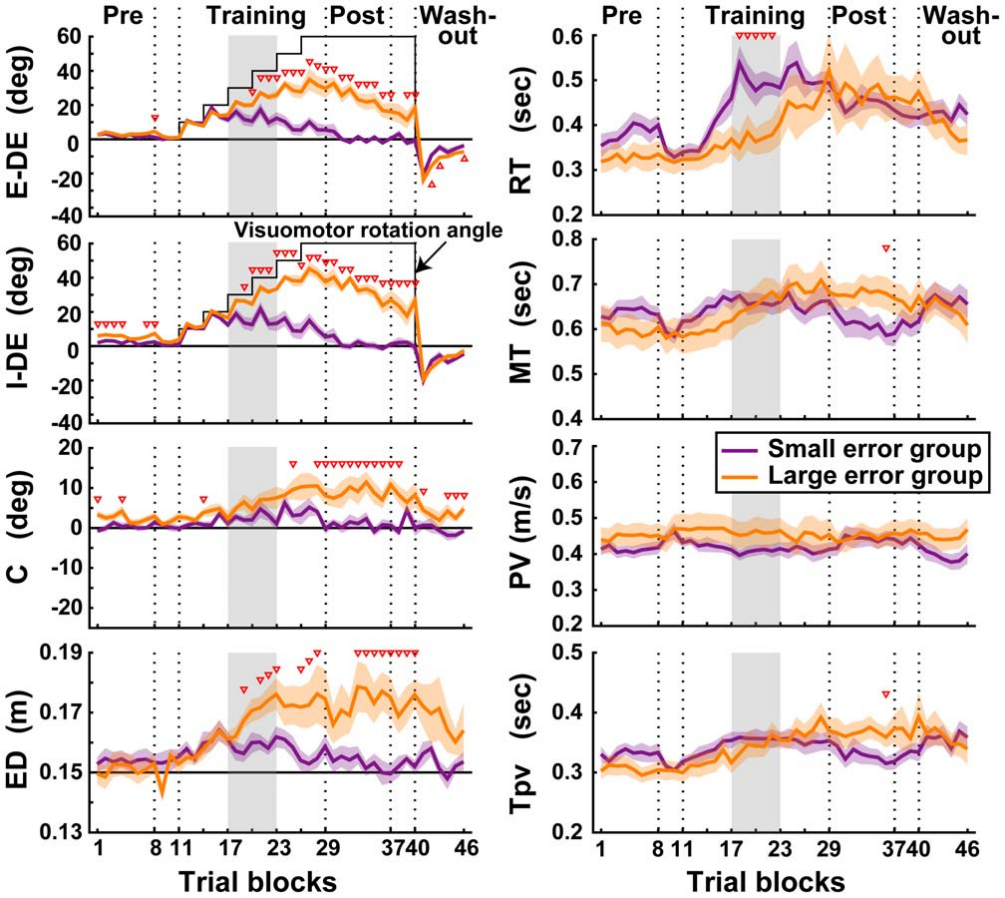
Changes in reaching movement characteristics by the gradual visuomotor rotation learning without online cursor feedback. Thick purple and orange lines in each panel indicate the SEG and LEG, respectively. Gray shaded blocks indicate the middle stage of the training phase (from the 18^th^ to 22^nd^ blocks, see Results). The notation is same as in Fig. 2A.

Differences in the mean characteristics indices between the subject groups are shown in Fig. 7. The trajectory errors (E-DE, I-DE, C, and ED) of the LEG (orange lines) increased progressively in the training phase. In the post-training phase, the E-DE and I-DE slightly decreased but did not return to the baseline. On the other hand, the trajectory errors of the SEG (purple lines) returned to the baseline until the post-training phase.

Here, we focused on the difference in the RT between the groups. The RT in the middle stage of the training phase was different between the groups. From the 18^th^ to 22^nd^ blocks (gray shaded blocks in the training phase in Fig. 7), the RT in the SEG was significantly longer than that in the LEG (*t* test, *p*<0.05). From this stage, the E-DE, I-DE, and ED in the SEG progressively decreased. Therefore, the increase in the RT in the SEG may be associated with the decrease in the trajectory error.

In the LEG, on the other hand, the E-DE, I-DE, C, and DS continuously increased in the middle stage of the training phase. However, the E-DE and I-DE were significantly smaller than the visuomotor rotation angle in the middle stage (unpaired *t* test, *p*<0.05 from the 18^th^ to 22^nd^ blocks). This indicates that in the middle stage of the training phase, the reaching movement slowly but significantly adapted to the visuomotor rotation with little increase in the RT. In the late stage of the training, the E-DE and I-DE decreased with the RT prolongation. Additionally, the C and DS did not increase compared with those in the middle stage of the training. Therefore, as in the SEG, the increase in the RT at the late stage of the training may be associated with the decrease in the trajectory error.

Taken together, the above observations suggest that the large reduction of the reaching error was associated with the increase in RT. In addition, the gradual I-DE decrease without RT change observed in the middle stage of the LEG training phase indicates that the reaching error can be reduced without the increase in RT.

## Discussion

### Change in Motor Learning Strategy According to Training Condition

By investigating the reaching adaptations to suddenly and gradually introduced visuomotor rotations, we have found several differences in the behavioral features among the training conditions after the learning. Here, we examined the qualitative differences in the visuomotor learning process from the viewpoint of the computational mechanisms for the visually guided reaching shown in Fig. 1C.

In the sudden condition, the RT increased after the training, suggesting that the learning is associated with the change in the motor planning. Since a large and obvious reaching error was induced by the visuomotor rotation and RT subsequently increased (Fig. 2A), the increase in RT would be associated with the awareness of the visuomotor discrepancy. In addition, the hand movements in the post-training phase were very similar to those before the pre-training phase not only in the trajectory but also in the temporal pattern of the movement (e.g., MT, PV, and Tpv). Therefore, these results suggest that in the sudden condition, the subjects might have shifted the hand movement direction relative to the target direction based on the awareness of the visuomotor discrepancy. Actually, a previous study [31] has demonstrated that such a “mental rotation” of the imagined movement vector about its origin leads to increased RT. In the early stage of the training, the reaching errors did not abruptly return to the baseline. Since the subjects were not informed of the presence of the visuomotor rotation, they might have searched for the appropriate motor planning to move the cursor to the target position at this stage and then might have found the valid mental rotation.

Note that, the RT increase after the learning in the sudden condition may not rule out the adaptation of the controllers. A previous study [32] has shown that explicit instruction given to the subjects quickly reduces the reaching error but does not suppress the implicit visuomotor adaptation. In their experiment, the subjects were instructed to move the hand in the shifted direction from the target direction, which may have led to the mental rotation of the desired movement. Their key finding was that the automatic adaptive process, which may correspond to the controller in our computational scheme, is not suppressed even if an explicit learning task is imposed.

In the gradual condition, on the other hand, the RT was not significantly altered by the rotation learning (green lines in Fig. 2A). Although the I-DE in the training phase of the gradual condition progressively increased, the I-DE was smaller than the visuomotor rotation angle. This suggests that the feedforward controller would slowly adapt to the gradual visuomotor rotation. Despite the increase in the I-DE, the trajectory curvature was increased and the E-DE was reduced with learning. Since the hand did not reach correctly in the catch trials (Fig. 3), online cursor feedback was required in order to successfully arrive at the target. Furthermore, the increase in the gain of visual feedback control after the training (Fig. 4C) suggests that the online visual feedback control would adapt to effectively compensate for the reaching error. Previous studies have suggested that the online adjustment of the reaching is involuntarily induced by the online visual feedback information [10], [17], [33]. Therefore, the reaching error in the gradual condition would be reduced involuntarily by the online visual feedback controller and the motor planning might therefore not be changed unlike in the sudden condition.

In previous studies, however, the gradually introduced visuomotor rotation did not result in a curved trajectory or a significant increase in the I-DE [7], [34], [35]. One possible explanation for this difference is the discrepancy in rate of increase of the angle of visuomotor rotation. In our experiment, the angle of the visuomotor rotation increased 10° every three trials in one target direction in the gradual condition. On the other hand, in Kagerer's experiment [7], for example, it increased 10° every 15 trials in one target direction and the I-DE did not significantly increase with learning. Therefore, in our setup, the rotation angle might increase before the I-DE is returned to the baseline by the slow adaptation of the feedforward controller. This point should be experimentally clarified in future study.

In contrast to the gradual condition of experiment 1, the learning of the gradual visuomotor rotation without online cursor feedback (experiment 2) led to an increase in the RT with a decrease in the feedback gain (Fig. 5). This suggests that the gradual visuomotor rotation does not simply lead to the learning without the change in the motor planning, whereas the learning strategy is changed by the manner of visual feedback. To reduce the reaching error without the online visual feedback, the motor planning could be changed even if the rotation angle is gradually increased.

In experiment 2, we divided the subjects into two groups in terms of the E-DE in the post training phase; the SEG of which the subjects could reach correctly and the LEG of which the subjects could not (Fig. 6). Then we found a difference in the learning strategy between the groups. In the SEG, the RT increased and the reaching error decreased in the middle stage of the training phase, suggesting that the motor planning would change to reduce the reaching error at this stage. In the LEG, on the other hand, the reaching error still increased at this stage with the less prolongation of the RT than that in the SEG. However, the I-DE in the LEG was significantly smaller than the visuomotor rotation angle, suggesting that the feedforward controller would slowly adapt to the rotation. Subsequently, the RT increased from the late stage of the training phase and the reaching error was reduced. This suggests that in the LEG, the feedforward controller first adapted to the visuomotor rotation and then the motor planning was changed to reduce the reaching error in the late stage of the training phase In other words, the initiation time of the change in the motor planning in the LEG training phase would be delayed compared to that in the SEG training phase. The reaching error difference after the learning may therefore be due to the difference in the learning strategy between the groups, although we need further investigation to determine what factor induced the learning-strategy difference.

Taken together, the above observations suggest that the reaching movement error caused by the slow adaptation of the feedforward controller would be reduced by changing the motor planning and by correcting the ongoing movement. This implies that the adaptations of motor planning and feedback controller would be more useful for quickly reducing the reaching error than the feedforward controller adaptation. Multiple adaptive-processes with different timescales have been discussed in several studies [5], [36]. The change in the learning strategy which enables different timescale adaptation might be required in order to effectively reduce the reaching error depending on the training condition.

### Visuomotor Learning Strategy Affects Size of Aftereffect

As in a previous study [7], the training in the gradual condition led to a larger aftereffect than that in the sudden condition in experiment 1. Considering that the change in the training conditions would lead to a change in learning strategy, the aftereffect difference between the conditions appears to reflect not only the extent of the adaptation but also the difference in the learning strategy.

We focused on the I-DE to compare the aftereffects between the training conditions, because, as mentioned above, the I-DE would not be affected by the online visual feedback control. Therefore, on the basis of the computational scheme we posited above (Fig. 1C), we consider that the I-DE aftereffect would reflect both of the adaptations of the motor planning and feedforward controller.

As shown in Figs. 2A and 7, the size of the I-DE aftereffect would be related to the RT change during learning. In the sudden condition of experiment 1 and the gradual condition of experiment 2, the RT significantly increased with learning and the small I-DE aftereffect was observed. In the gradual condition of experiment 1, in contrast, the RT did not change and the large I-DE aftereffect was observed. Since the RT increase would be related to the change in the motor planning as discussed above, these results suggest that the size of the I-DE aftereffect would be associated with the learning strategy with or without the change in the motor planning. The learning strategy with changing motor planning might quickly reduce the I-DE aftereffect in the washout phase. On the other hand, the learning strategy without changing the motor planning might slowly reduce the I-DE aftereffect because the I-DE would be reduced by the slow adaptation of the feedforward controller.

Considering that in the gradual condition of experiment 1, as discussed above, the slow I-DE reduction would be related to the adaptation of the feedforward controller, the negative correlation between the size of the I-DE aftereffect and the increase in the gain of the visual feedback control after the gradual rotation learning (Fig. 4D) could be interpreted as a trade-off between the adaptations of the feedforward and feedback controllers: if the feedforward controller adapts predominantly, the feedback controller does not adapt very much, and vice versa. The contribution ratio between the feedforward and feedback controller adaptations varied among the subjects in the gradual condition, and the adaptation of the feedforward controller would lead to the large I-DE aftereffect. Our experimental data therefore suggest that differences in the motor learning strategies due to training conditions and inter-subject variation result in the different motor performance in visuomotor transformation.

## References

[1] Cunningham HA (1989). Aiming error under transformed spatial mappings suggests a structure for visual-motor maps.. J Exp Psychol Hum Percept Perform.

[2] Ghahramani Z, Wolpert DM, Jordan MI (1996). Generalization to local remappings of the visuomotor coordinate transformation.. J Neurosci.

[3] Imamizu H, Uno Y, Kawato M (1995). Internal representations of the motor apparatus: implications from generalization in visuomotor learning.. J Exp Psychol Hum Percept Perform.

[4] Kitazawa S, Kohno T, Uka T (1995). Effects of delayed visual information on the rate and amount of prism adaptation in the human.. J Neurosci.

[5] Krakauer JW, Pine ZM, Ghilardi MF, Ghez C (2000). Learning of visuomotor transformations for vectorial planning of reaching trajectories.. J Neurosci.

[6] Vetter P, Goodbody SJ, Wolpert DM (1999). Evidence for an eye-centered spherical representation of the visuomotor map.. J Neurophysiol.

[7] Kagerer FA, Contreras-Vidal JL, Stelmach GE (1997). Adaptation to gradual as compared with sudden visuo-motor distortions.. Exp Brain Res.

[8] Michel C, Pisella L, Prablanc C, Rode G, Rossetti Y (2007). Enhancing visuomotor adaptation by reducing error signals: single-step (aware) versus multiple-step (unaware) exposure to wedge prisms.. J Cogn Neurosci.

[9] Desmurget M, Grafton S (2000). Forward modeling allows feedback control for fast reaching movements.. Trends Cogn Sci.

[10] Gomi H (2008). Implicit online corrections of reaching movements.. Curr Opin Neurobiol.

[11] Kawato M (1999). Internal models for motor control and trajectory planning.. Curr Opin Neurobiol.

[12] Sabes PN (2000). The planning and control of reaching movements.. Curr Opin Neurobiol.

[13] Wolpert DM, Ghahramani Z (2000). Computational principles of movement neuroscience.. Nat Neurosci.

[14] Wolpert DM, Miall RC, Kawato M (1998). Internal models in the cerebellum.. Trends in Cognitive Sciences.

[15] Saijo N, Gomi H (2009). Motor Learning Strategies for Suddenly and Gradually Increased Visuomotor Rotations.. IEICE TRANSACTIONS on Information and Systems (in Japanese).

[16] Gomi H, Kawato M (1996). Equilibrium-point control hypothesis examined by measured arm stiffness during multijoint movement.. Science.

[17] Saunders JA, Knill DC (2004). Visual feedback control of hand movements.. J Neurosci.

[18] Sober SJ, Sabes PN (2003). Multisensory integration during motor planning.. J Neurosci.

[19] Schaal S, Arbib MA (2002). Learning robot control.. The handbook of brain theory and neural networks, 2nd Edition. 2 ed.

[20] Chen-Harris H, Joiner WM, Ethier V, Zee DS, Shadmehr R (2008). Adaptive control of saccades via internal feedback.. J Neurosci.

[21] Gomi H, Kawato M (1992). Adaptive feedback control models of the vestibulocerebellum and spinocerebellum.. Biol Cybern.

[22] Gomi H, Kawato M (1993). Neural network control for a closed-loop System using Feedback-error-learning.. Neural Networks.

[23] Miall RC, Weir DJ, Wolpert DM, Stein JF (1993). Is the cerebellum a smith predictor?. J Mot Behav.

[24] Flanagan JR, Wing AM (1997). The role of internal models in motion planning and control: evidence from grip force adjustments during movements of hand-held loads.. J Neurosci.

[25] Ghez C, Gordon J, Ghilardi MF (1995). Impairments of reaching movements in patients without proprioception. II. Effects of visual information on accuracy.. J Neurophysiol.

[26] Gomi H, Shidara M, Takemura A, Inoue Y, Kawano K (1998). Temporal firing patterns of Purkinje cells in the cerebellar ventral paraflocculus during ocular following responses in monkeys I. Simple spikes.. J Neurophysiol.

[27] Gordon J, Ghilardi MF, Ghez C (1995). Impairments of reaching movements in patients without proprioception. I. Spatial errors.. J Neurophysiol.

[28] Gribble PL, Ostry DJ (1999). Compensation for interaction torques during single- and multijoint limb movement.. J Neurophysiol.

[29] Shidara M, Kawano K, Gomi H, Kawato M (1993). Inverse-dynamics model eye movement control by Purkinje cells in the cerebellum.. Nature.

[30] Yamamoto K, Kawato M, Kotosaka S, Kitazawa S (2007). Encoding of movement dynamics by Purkinje cell simple spike activity during fast arm movements under resistive and assistive force fields.. J Neurophysiol.

[31] Georgopoulos AP, Massey JT (1987). Cognitive spatial-motor processes. 1. The making of movements at various angles from a stimulus direction.. Exp Brain Res.

[32] Mazzoni P, Krakauer JW (2006). An implicit plan overrides an explicit strategy during visuomotor adaptation.. J Neurosci.

[33] Franklin DW, Wolpert DM (2008). Specificity of reflex adaptation for task-relevant variability.. J Neurosci.

[34] Klassen J, Tong C, Flanagan JR (2005). Learning and recall of incremental kinematic and dynamic sensorimotor transformations.. Exp Brain Res.

[35] Yamamoto K, Hoffman DS, Strick PL (2006). Rapid and long-lasting plasticity of input-output mapping.. J Neurophysiol.

[36] Smith MA, Ghazizadeh A, Shadmehr R (2006). Interacting adaptive processes with different timescales underlie short-term motor learning.. PLoS Biol.

